# Applying the WHO ICF Framework to Fetal Alcohol Spectrum Disorder (FASD): A Forensic and Clinical Perspective on Disability Assessment and Patient Support

**DOI:** 10.3390/healthcare13192546

**Published:** 2025-10-09

**Authors:** Davide Ferorelli, Francesco Calò, Gianmarco Sirago, Dania Comparcini, Filippo Gibelli, Francesco Sessa, Marco Carotenuto, Biagio Solarino, Monica Salerno

**Affiliations:** 1 Section of Legal Medicine, Interdisciplinary Department of Medicine (DIM), University of Bari “Aldo Moro”, 70124 Bari, Italy; davide.ferorelli@uniba.it (D.F.); gianmarcosirago@gmail.com (G.S.); dania.comparcini@uniba.it (D.C.); biagio.solarino@uniba.it (B.S.); 2 Department of Medical, Surgical and Advanced Technologies “G.F. Ingrassia”, University of Catania, 95121 Catania, Italy; filippogibellimedico@gmail.com (F.G.); francesco.sessa@unict.it (F.S.); monica.salerno@unict.it (M.S.); 3 Clinic of Child and Adolescent Neuropsychiatry, Department of Mental Health, Physical and Preventive Medicine, University of Campania “Luigi Vanvitelli”, 81100 Caserta, Italy; marco.carotenuto@unicampania.it

**Keywords:** FASD, epigenetics, neurodevelopment, ICF, social impairment, quality in healthcare

## Abstract

**Background/Objectives**: This article aims to investigate the multifaceted effects of alcohol on neurophysiopathological development from gestational stages through adult life and the consequent dynamic-relational challenges in individuals with Fetal Alcohol Spectrum Disorder (FASD). FASD, resulting from prenatal alcohol exposure (PAE), is characterized by a range of neurological, cognitive, behavioral, and sometimes physical impairments. This article explores how alcohol and its toxic metabolites cross the placenta, inducing direct cellular toxicity and epigenetic alterations that disrupt critical neurodevelopmental processes such as neurogenesis and brain circuit formation. Clinically, individuals with FASD exhibit diverse deficits in executive functioning, learning, memory, social skills, and sensory-motor abilities, leading to significant lifelong disabilities. A central focus is the application of the World Health Organization’s International Classification of Functioning, Disability and Health (ICF) criteria to comprehensively frame these disabilities. The ICF’s biopsychosocial model allows for a multidimensional assessment of impairments in body functions and structures, limitations in activities, and restrictions in participation, while also considering the crucial role of environmental factors. **Methods**: PubMed and Semantic Scholar databases were searched for relevant papers published in English. **Results**: This article highlights the utility of the ICF in creating individualized functioning profiles to guide interventions and support services, addressing the limitations of traditional assessment methods. **Conclusions**: While the ICF framework offers a robust approach for understanding and managing FASD, further research is essential to develop and validate FASD-specific ICF-based assessment tools to enhance support and social participation for affected individuals.

## 1. Introduction

### 1.1. Definition

Fetal Alcohol Spectrum Disorder (FASD), classified in ICD-9 under codes 655.4 and 760.71, is a neurodevelopmental condition characterized by a broad range of cognitive and behavioral impairments, often associated with neurological alterations. In some cases, these deficits may be accompanied by craniofacial anomalies, growth retardation, and congenital abnormalities. FASD results from prenatal alcohol exposure and encompasses several diagnostic entities, including Fetal Alcohol Syndrome (FAS) and Partial Fetal Alcohol Syndrome (pFAS) [1,2].

### 1.2. Molecular and Genetic Aspects

Ethanol’s biological effects are closely related to its metabolism. The primary enzyme responsible for the initial oxidative metabolism of ethanol is cytosolic alcohol dehydrogenase (ADH), which converts it into acetaldehyde, a hydrophilic molecule, and produces reduced nicotinamide adenine dinucleotide (NADH). A secondary pathway, accounting for roughly 10% of ethanol metabolism, involves the microsomal cytochrome P450 (CYP) system. While CYP1A2 and CYP3A4 play a role, CYP2E1 is considered the main CYP enzyme involved. This oxidative process occurs within the endoplasmic reticulum of liver cells, where CYP2E1, utilizing NADPH and oxygen, transforms ethanol into acetaldehyde, which is then further converted to acetate. Notably, this conversion of ethanol to acetaldehyde generates reactive oxygen species (ROS), which are significant contributors to alcohol’s toxicity. The acetaldehyde produced through these pathways is subsequently oxidized to acetate by aldehyde dehydrogenase (ALDH). Chronic alcohol consumption is known to decrease ALDH activity, leading to elevated acetaldehyde levels. The activity levels of different ADH and ALDH isoforms influence acetaldehyde concentrations and are considered a risk factor for developing alcoholism. Indeed, symptoms of ethanol intolerance, such as nausea, difficulty swallowing, headaches, and especially facial flushing caused by vasodilation, are linked to acetaldehyde concentration [3].

From an epidemiological perspective, individuals with FASD are identified as those with confirmed or suspected prenatal alcohol exposure (PAE).

In the specific case of FASD, during the gestational period, the fetus and mother “share” the same circulatory system, the fetus being supported in its development by the vascular supply provided by the pregnant woman through the vascular-placental system, which is incapable of operating a protective “filter” action against the ethanol molecule and its toxic metabolites. Evidence of placental permeability to substances dates back considerably, with figures like J.W. Ballantyne recognizing conditions like “fetal alcoholism” as early as 1904 [4]. Exposure of the fetus and placenta to various substances can have a wide range of outcomes, from minimal impact to structural or neurodevelopmental differences, or even pregnancy loss, and the consequences are often unpredictable. Passive diffusion is the main mechanism for many drugs to cross the placenta, particularly hydrophobic molecules under 600 Da, while hydrophilic molecules, such as alcohol, require carrier proteins. The human placenta contains enzymes for both phase I (oxidation, reduction, hydrolysis) and phase II (conjugation) substance metabolism, including alcohol dehydrogenases. The severity and duration of a substance’s effects on the fetus are determined by the maternal dose, maternal and fetal substance processing (pharmacokinetics), and individual factors such as genetic variations in substance metabolism (pharmacogenetics), existing health conditions, and other medications being taken. Another critical factor influencing fetal substance exposure is how quickly and extensively the substance distributes across the placenta. Some substances cross the placental membranes rapidly, while others distribute more slowly, potentially due to limited capacity carrier-mediated uptake. However, with repeated or continuous substance administration, the latter can accumulate in the fetus over time, potentially leading to significant lasting effects [5].

The development of a complex organism from a single cell, characterized by diverse, interactive, and responsive cell types, is an inherently intricate process. The human brain’s development, beginning early in gestation and extending through adolescence, is particularly complex and remains incompletely understood. Each stage of this developmental journey is precisely controlled by gene expression, regulated in a time- and location-specific manner. This delicate process is susceptible to influence from external factors, including maternal gene products, the availability of micronutrients, and environmental molecules. Epigenetic processes, which alter chromatin structure without changing the underlying DNA sequence, are crucial regulators of gene expression. These mechanisms, such as DNA methylation and post-translational modifications of histone proteins, play a vital role in numerous cellular functions. Specifically, dynamic methylation and demethylation of DNA within individual genes in the central nervous system are critical for proper synaptic and cognitive function. Additional gene regulatory mechanisms include microRNAs. Significantly, alcohol-induced alterations to these epigenetic processes can profoundly impact both normal developmental and adult brain gene expression. Alcohol consumption during pregnancy, known as prenatal alcohol exposure (PAE), induces various epigenetic changes associated with harmful effects on the developing fetus. To understand how PAE leads to the clinical findings related to FASD observed in human populations, it is essential to decipher how alcohol affects the molecular processes that guide neurodevelopment. Studies have demonstrated that ethanol exposure at different stages of prenatal development disrupts fundamental processes like neurogenesis, neuronal communication, and the formation of neural circuits. These disruptions occur through changes in key molecular mechanisms, including alterations in DNA methylation patterns, aberrant post-translational modifications of histone proteins, and abnormalities in microRNA regulation. Exposure to a high dose of ethanol, characteristic of “binge” drinking mothers, is particularly detrimental. When this occurs at specific points during development (such as the mid-first, second, or third trimester), it can critically disrupt the molecular processes essential for that stage of brain development, leading to long-term physical and behavioral abnormalities. Ethanol, especially at high concentrations, is directly toxic to cells undergoing active developmental processes such as neurulation, differentiation, migration, and synaptogenesis. While apoptosis is a normal developmental process for removing excess or underperforming neurons, alcohol can inappropriately induce this process in the developing brain by acting as an NMDA receptor antagonist and a GABAergic agonist, interfering with critical signaling pathways. Analysis of gene expression profiles following ethanol exposure reveals shifts towards increased expression of genes associated with apoptosis and cell membrane integrity, while genes involved in cell division and molecule synthesis show decreased expression. This pattern suggests that ethanol exposure triggers a strong stress response in developing cells, potentially aimed at reducing energy-intensive processes to enhance survival. A single high-dose exposure can trigger widespread programmed cell death (apoptosis) in vulnerable cell types within the developing brain. This phenomenon has been observed across multiple brain regions and developmental stages. The vulnerability of brain areas varies with timing; for instance, derivatives of the ventromedial forebrain and gastrulating mesodermal cells show vulnerability early (equivalent to the first trimester), while others, including the hippocampus, cerebellum, corpus callosum, and parts of the prefrontal cortex, are sensitive much later (equivalent to the third trimester).

In this regard, important factors that can influence the impact of prenatal alcohol exposure include the dose and developmental timing in which the exposure occurs. In terms of dosage, the amount of alcohol to which the fetus is exposed influences the extent of the damage and severity of developmental deficits [6]. Neuroimaging studies consistently reveal structural brain abnormalities in individuals exposed to alcohol prenatally. These include reduced overall brain and cerebellar size, incomplete development of the corpus callosum, and smaller volumes in the hippocampus, caudate nucleus, and basal ganglia. Furthermore, individuals without the characteristic facial features of FASD often show increased regional cortical thickness and gray matter, alongside decreased volume and disorganization in white matter. Diffusion tensor imaging provides further evidence, indicating reduced connectivity both within and between brain regions in individuals with PAE, significantly affecting structures like the corpus callosum, nerve tracts connecting to the frontal, occipital, and parietal lobes, and pathways involving the hypothalamic–pituitary–adrenal (HPA) axis. Altered trajectories of neurodevelopment during this critical 5–15 year-old period, particularly in frontal lobe connections, have been investigated in MRI scans and may further compound existing deficits and contribute to high rates of secondary disabilities that emerge during adolescence in FASD [7]. Despite the toxicity, not all developing neurons succumb to ethanol. Certain cell types that encounter this harmful environment undergo molecular adaptation and continue to develop. These surviving cells can proceed through further division and differentiation, establishing synaptic connections and integrating into the mature brain’s functional network, although their development may be altered [8,9].

### 1.3. Clinical Manifestations

The typical symptoms, attributable to neurological deficit, are behavioral alterations. Individuals affected by FASD show deficits in abstract thinking, organization, planning, learning, remembering sequences of events, connecting cause-and-effect relationships, expressive and receptive language, social skills, and awareness and regulation of behaviors and emotions. They have difficulty learning and retaining new information, recognizing contextual relationships, and following rules. This symptomatology, comparable to an attention deficit disorder, creates difficulties in maintaining personal organization and in carrying out assigned tasks, not only school-related but also recreational [10,11]. At the same time, like adults who chronically consume alcohol, a phenomenon of chronic sleep disorder may begin to manifest, further compromising the performance of daily activities. Their behavior, due to alcohol-related brain injury, may sometimes be misinterpreted as intentional. However, children with FASD are often eager to engage with others but may be more vulnerable to peer influence and exploitation and may have difficulty assessing the social consequences of their actions, being too trusting of other children and even strangers. Consequently, they often find themselves in unpleasant situations that put them at a disadvantage. Individuals who have sustained central nervous system damage due to prenatal alcohol exposure frequently exhibit a spectrum of disabilities. These range from difficulties in social interaction to, in more severe instances, compromised abilities in decision-making, self-determination, and discerning the societal implications of their actions. Such impairments may increase vulnerability to difficulties with decision-making and, in some cases, to involvement with the justice system [12,13]. Moreover, often organic neurological-sensory damage is present: visual deficits (optic nerve hypoplasia, retinal alterations from vascular damage, convergent strabismus) and/or auditory deficits (both conductive and sensorineural hearing loss) that further aggravate the problems in the learning and dynamic-relational spheres. Also in this area, impairments can arise from deficits in both gross motor functions (balance and coordination) and fine motor functions (dysgraphia). A small percentage may also be affected by epileptogenic seizures [1,14,15].

### 1.4. Diagnosis and Therapeutic Challenges

There are no specific drug treatments for FASD, and clinicians often use combinations of medications for ADHD, disorders of impulse control, aggression, and mood disorders. The diagnosis is based on international guidelines, with diagnostic criteria including facial features, delayed growth, structural and/or functional disorders of the central nervous system (CNS), and prenatal alcohol exposure. The most widely used are the guidelines of the Institute of Medicine (IOM) of the National Academy of Sciences of the United States, the guidelines of the Centers of Disease Prevention and Control (CDC), the 4-digit diagnostic code of the University of Washington (Astley and Warren), and the Canadian guidelines (Table 1) [1,16,17]. The importance of early diagnosis is underscored by the fact that any delay in social support makes the consequences of FASD irreparable [12,18].

### 1.5. International Classification of Functioning (ICF)

Regarding the multidimensional assessment proposed by this legislative decree, it suggests the application of the International Classification of Functioning, Disability, and Health (ICF), adapted from rehabilitation medicine, or the World Health Organization Disability Assessment Schedule (WHODAS) contexts. Functioning and disability, within this framework, are further divided into (i) body functions and structures (BFS) and (ii) activities and participation. The ICF framework describes how personal, social, and environmental factors impact the individual’s disability and functioning [19]. So, regarding the ICF, it evaluates several factors. The individual presents several functional impairments and contextual factors according to the ICF framework. At the level of body functions and structures, there are significant deficits in cognitive functions, including impaired attention, memory, learning capacity, problem-solving, and executive control (b1). Sensory functions are also compromised, particularly in visual, auditory, or multisensory integration domains (b2). Difficulties in voice and speech functions are evident, affecting articulation and fluency (b3). Furthermore, there may be comorbidities involving the cardiovascular, respiratory, immune, endocrine, and metabolic systems (b4) (Table 2). In terms of activities, the individual struggles with acquiring and applying knowledge, including foundational literacy skills such as reading and writing (d1). Motor difficulties affect both fine and gross movements, resulting in reduced mobility (d4). There are also limitations in performing domestic activities (d6), as well as challenges in initiating and maintaining interpersonal relationships (d7) (Table 3). Regarding environmental factors, the level and quality of available support—educational, therapeutic, or familial—play a critical role (e3). Additionally, societal attitudes can either facilitate or hinder inclusion, with stigmatizing perceptions acting as a significant barrier (e4) (Table 4) [20].

## 2. Materials and Methods

This article aims to synthesize current knowledge on the biological, clinical, and social consequences of prenatal alcohol exposure, with particular emphasis on disability conceptualized through the WHO International Classification of Functioning, Disability and Health (ICF). To identify relevant studies, a literature search was conducted through a search of PubMed, Scopus, and Web of Science databases. The strategy combined controlled vocabulary and free-text terms; exemplary strings were (“fetal alcohol spectrum disorder” OR “prenatal alcohol exposure”) AND (ICF OR “International Classification of Functioning”). Limits were humans and the English language. Eligible records included articles, reviews, and clinical guidelines that reported biological or epigenetic mechanisms, neurodevelopmental, cognitive, behavioral, or structural CNS outcomes of PAE/FASD, or that assessed disability and functioning. We excluded case reports, narrative commentaries, conference abstracts, animal or in vitro studies, and articles without the aforementioned topics.

Moreover, we operationalized the six SANRA items as follows: topic rationale grounded in the public-health and medico-legal relevance of FASD; clear statement of purpose focused on synthesizing biological, clinical, and ICF-based disability implications; literature coverage through structured searches using controlled vocabulary and free-text terms related to prenatal alcohol exposure, FASD, disability, and ICF; reference use privileging peer-reviewed primary studies, guidelines, and high-quality reviews with consistent citation formatting; logical flow with an ICF-oriented synthesis in Results and a practice-focused Discussion; and presentation of evidence with explicit study types and clinically relevant endpoints when available. The completed checklist is provided as Table A1 in the Appendix A section of the present article [21].

## 3. Results

Young adults with FASD experience a wide range of disabilities affecting multiple domains of functioning. According to data from the Canadian National FASD Database, adolescents, transition-aged youth, and adults with FASD present with high rates of difficulties in daily living, legal problems with offending (30%), assisted or sheltered housing (21%), and school disruption [22,23]. Children with fetal alcohol spectrum disorders (FASD) may be at a higher risk of involvement in child welfare and thus out-of-home placement [24].

The cognitive and behavioral impairments associated with FASD can lead to significant challenges in social interactions, education, and employment. Adults with FASD are less likely to be in relationships and more likely to have poor educational outcomes compared to their peers without FASD.

When analyzed using the WHO ICF framework, FASD-related impairments can be categorized across multiple domains of functioning [25]. Body functions affected by FASD include cognitive functions (b1), particularly global intellectual functions (b117), and specific mental functions such as attention (b140), memory (b144), and higher-level cognitive functions (b164). Sensory functions and pain (b2) may also be affected, as evidenced by visual impairments and a thinner retinal nerve fiber layer found in young adults with FASD.

Activities and participation restrictions in individuals with FASD include difficulties in learning and applying knowledge (d1), general tasks and demands (d2), communication (d3), self-care (d5), domestic life (d6), interpersonal interactions and relationships (d7), major life areas such as education (d810–d839) and employment (d840–d859), and community, social, and civic life (d9). Research has shown that young adults with FASD experience significant challenges in these areas, as evidenced by high rates of unemployment, housing instability, and difficulty with independent living [23].

Environmental factors play a crucial role in either facilitating or hindering functioning and participation for individuals with FASD. Supportive environments, including family support, appropriate educational accommodations, and access to specialized services, can significantly improve outcomes for individuals with FASD. Conversely, unsupportive environments, social stigma, and lack of awareness about FASD among service providers can exacerbate difficulties and lead to adverse outcomes [26].

The creation of functioning profiles using the ICF framework can guide interventions and environmental adaptations for individuals with FASD. By identifying specific limitations in body functions, activities, and participation, as well as relevant environmental factors, these profiles provide a comprehensive picture of an individual’s needs and potential areas for intervention.

The International Classification of Functioning for Children and Youth (ICF-CY) builds upon the ICF framework to specifically address factors supporting the growth, health, and development of children and youth with disabilities. Within the ICF model, “activity” and “participation” are considered together. Activity refers to an individual’s ability to perform a task or action, evaluated in terms of both actual performance and capacity. The concept of participation, however, is viewed as a more complex and evolving idea that extends beyond the ICF definition. It encompasses the personal significance, values, and experiences of individuals and includes the “complex, socially embedded, and personally meaningful life roles and activities that children undertake”. [19]

Children with FASD who also experience motor coordination difficulties may face challenges with activities and participation in daily life at home, such as getting dressed or bathing. These difficulties can manifest as slower and clumsier task execution, often requiring assistance from caregivers. Furthermore, the various neurodevelopmental impairments associated with FASD can hinder their ability to participate in everyday activities across different settings, including home, school, and the community [1,14,19]. Moreover, up to 60% of individuals with FASD have been in conflict with the law, often facing charges for property-related or interpersonal offenses [12,27].

Support needs for young adults with FASD vary depending on the severity of impairment, individual strengths and challenges, and environmental factors. Research has shown that many individuals with FASD require high levels of support in areas such as independent living, employment, education, and social relationships. Early assessment, diagnosis, and intervention are critical protective factors against adverse outcomes in FASD [23,28].

## 4. Discussion

The definition of disability, borrowed from the WHO’s enunciations, defines it as “enduring physical, mental, intellectual or sensory impairments that, in interaction with barriers of various kinds, may hinder full and effective participation in the various contexts of life on an equal basis with others”.

### 4.1. ICF’s Contribution

The application of the WHO ICF framework to FASD provides a comprehensive understanding of the condition by considering not only the impairments in body functions and structures but also the limitations in activities, restrictions in participation, and the impact of environmental factors. This multidimensional and biopsychosocial approach highlights the complex interaction between the individual with FASD and their environment, which can either facilitate or hinder functioning and participation. The strength of the ICF lies in its ability to integrate health conditions, personal factors, and environmental influences, allowing for a detailed and individualized assessment of functioning and disability that is particularly valuable for heterogeneous conditions such as FASD [29].

The findings also emphasize the importance of considering the developmental trajectory of FASD. As children with FASD transition to adolescence and young adulthood, they face new challenges related to independence, education, employment, and social relationships. The difficulties experienced by young adults with FASD signal a high level of service needs that must be addressed through appropriate support and intervention [23].

This approach allows for a more nuanced understanding of each individual’s situation and can guide the development of personalized support plans [28].

### 4.2. Examples of Rehabilitation/Forensic Practice

The findings of this article have significant implications for understanding the long-term consequences of FASD in young adults. FASD creates lifelong physical, mental, cognitive, and behavioral deficits that impact many aspects of daily living. The high rates of difficulties in daily living, including independent living support needs, substance misuse, employment problems, and legal issues, underscore the pervasive and enduring nature of FASD-related disabilities.

The high rates of difficulties in daily living indicate a need for comprehensive and coordinated services across multiple domains. Ongoing functional and needs-based service provision is essential as youth with FASD transition to adulthood and beyond. Support services should address the specific challenges faced by individuals with FASD, including cognitive impairments, difficulties with social skills and emotional regulation, and challenges in education and employment [23].

Early assessment, diagnosis, and intervention are critical protective factors against adverse outcomes in FASD. Ongoing functional and needs-based service provision is essential as youth with FASD transition to adulthood and beyond. Support services should address the specific challenges faced by individuals with FASD, including cognitive impairments, difficulties with social skills and emotional regulation, and challenges in education.

Research consistently demonstrates that individuals with FASD are disproportionately represented in criminal justice systems. Neurocognitive impairments, such as deficits in executive functioning, memory, and adaptive behavior, contribute to difficulties in understanding legal procedures, increased vulnerability to peer influence, and higher risk of suggestibility [30]. These legal difficulties not only reflect individual neurodevelopmental impairments but also highlight systemic gaps, such as insufficient awareness of FASD among justice professionals and lack of appropriate forensic assessment protocols. Recent contributions suggest that integrating structured models, such as the Risk-Need-Responsivity framework, could help tailor interventions and reduce recidivism among justice-involved individuals with FASD.

In this regard, medico-legal training, with the acquired skills in the evaluation of damages in personal injury cases, can provide a contribution to the assessment of individuals affected by FASD, both at the clinical and social level [31].

### 4.3. Limitations and Research Agenda

The application of the WHO ICF to FASD is still emerging, and more research is needed to establish its validity and reliability in this population. The development and validation of assessment tools specifically designed for FASD, which incorporate the principles of the ICF and address the limitations of current methods, would be a significant contribution to the field [28]. Recent contributions suggest that integrating structured models, such as the Risk-Need-Responsivity framework, could help tailor interventions and reduce recidivism among justice-involved individuals with FASD [32].

## 5. Conclusions

FASD represents a significant public health concern with lifelong consequences for affected individuals. The neurological and systemic impairments associated with FASD lead to a wide range of disabilities that affect multiple domains of functioning, including cognition, social interaction, education, employment, and independent living. These disabilities persist into adulthood and require ongoing support and intervention.

There is also evidence of a strong association between FASD and heightened vulnerability to criminal justice involvement, driven by cognitive, behavioral, and social impairments. Individuals with FASD frequently encounter legal penalties that may not adequately account for their diminished culpability and heightened suggestibility. It is therefore essential that legal contexts adopt evidence-based approaches, including systematic screening for FASD, training of justice professionals, and the use of tailored intervention frameworks.

The assessment of disability in individuals with FASD presents unique challenges due to the heterogeneous nature of the condition and the limitations of the current assessment framework. The WHO ICF framework offers more comprehensive approaches to disability assessment that consider not only impairments in body functions but also limitations in activities, restrictions in participation, and the influence of environmental factors.

The findings of this paper highlight the need for a more individualized and multidimensional approach to disability assessment for individuals with FASD. The creation of functioning profiles using the ICF framework can guide interventions and environmental adaptations to improve functioning and quality of life. Early assessment, diagnosis, and intervention, combined with ongoing functional and needs-based service provision, are essential for mitigating the long-term impact of FASD and promoting social inclusion.

Future research should focus on developing and validating assessment tools specifically designed for FASD, evaluating the effectiveness of ICF-based interventions, and exploring the implementation of the WHO ICF framework. These efforts will contribute to improving the assessment and support of individuals with FASD, ultimately enhancing their quality of life and social participation.

## Figures and Tables

**Table 1:** Diagnostic guidelines for FASD assessment.

	IOM Criteria (2016)	CDC (2004)	C4-Digit Diagnostic Code (2000)	Canadian Guidelines (2015)
Facial features	2 of the following: - Palpebral fissure ≤ P10 - Smooth philtrum, rank 4 or 5 - Thin vermillion border, rank 4 or 5	3 of the following at any age: - Palpebral fissure < P3 - Smooth philtrum, rank 4 or 5 - Thin vermillion border, - rank 4 or 5	3 of the following at any age: - Palpebral fissure < P3 - Smooth philtrum, rank 4 or 5 - Thin vermillion border, rank 4 or 5	3 of the following at any age: - Palpebral fissure < P3 - Smooth philtrum, rank 4 or 5 - Thin vermillion border, rank 4 or 5
Neurodevelopmental impairment	Impairment in 1 or 2 of the following domains: - Motor skills - Neuroanatomy/neurophysiology - Cognition - Language - Academic performance - Memory - Attention - Executive function, including impulse control and hyperactivity - Affect regulation - Adaptive regulation, social skills or social communication	At least 1 of the following: (1) Structural/neurologic: e.g., HC < P10, abnormal structure, seizures, significant symptoms (2) Impairment: In 3 or more functional domains (1 or more SD below the mean) Global deficit (2 or more SD below the mean)	At least 1 of the following: (1) Structural/neurologic: e.g., HC < P3, abnormal structure, seizures, significant symptoms (2) Severe impairment: In 3 or more functional domains (2 or more SD below the mean)	Impairment in at least 3 of the following domains: - Motor skills - Neuroanatomy/neurophysiology - Cognition - Language - Academic performance - Memory - Attention - Executive function, including impulse control and hyperactivity - Affect regulation - Adaptive regulation, social skills or social communication
Impaired growth	Prenatal and/or postnatal weight and/or height and/or HC ≤ P10	Prenatal and/or postnatal weight or height < P10	Prenatal and/or postnatal weight or height < P10	(not considered)

**Table 2:** Body functions and structures (b) according to ICF.

ICF Code	Description
b1	Cognitive Functions: Difficulties in attention, memory, learning, problem-solving, and executive control.
b2	Sensory Functions: Problems with sight, hearing, or sensory integration.
b3	Voice and speech functions: articulation, fluency
b4	Cardiovascular, Respiratory, Immune, Endocrine, and Metabolic Functions: Possible associated medical complications.

**Table 3:** Activities (d) according to ICF.

ICF Code	Description
d1	Learning and applying knowledge:
d4	Mobility: Problems in fine and gross motor coordination.
d6	Domestic Activities: Limited skills in managing household tasks.
d7	Interpersonal Interactions: Challenges in social interactions and forming meaningful relationships.

**Table 4:** Environmental factors (e) according to ICF.

ICF Code	Description
e3	Support and Relationships: Access to support services and educational and therapeutic assistance.
e4	Attitudes: Inclusive or stigmatizing attitudes from the community.

## Data Availability

The original contributions presented in the study are included in the article. Further inquiries can be directed to the corresponding author.

## Abbreviations

The following abbreviations are used in this manuscript:

FASD	Fetal Alcohol Spectrum Disorder
pFASD	Partial Fetal Spectrum Disorder
PAE	Prenatal alcohol exposure
ICF	International Classification of Functioning, Disability and Health
WHODAS	World Health Organization Disability Assessment Schedule
WHO	World Health Organization
CYP	Microsomal cytochrome
NADH	Nicotinamide adenine dinucleotide
ADH	Alcohol dehydrogenase
ALDH	Aldehyde dehydrogenase
CDC	Centers of Disease Prevention and Control
IOM	Institute of Medicine
MRI	Magnetic Resonance Imaging
HC	Head circumference
P10	10th percentile
P3	3rd percentile
SD	Standard deviation

## Appendix A

**Table A1:** SANRA checklist.

SANRA Criterion	Score (0–2)	How This Article Meets the Criterion
Topic rationale	2	FASD is framed as a major public-health and medico-legal issue, justifying an ICF-based synthesis.
Purpose	2	The article aims to summarize biological mechanisms, clinical manifestations, and disability assessment within the WHO-ICF model.
Literature coverage	2	Searches conducted in PubMed, Semantic Scholar and Web of Science using terms on prenatal alcohol exposure, FASD, disability, ICF; references of key articles scanned.
Reference use	2	Peer-reviewed primary studies, guidelines, and reviews are prioritized and consistently cited.
Logical flow	2	Mechanisms are linked to clinical phenotypes and mapped to ICF domains.
Presentation	1	Study designs and clinically relevant endpoints are stated when available; findings are presented clearly.
Total	11/12	
